# Retraction Note: Serum bilirubin levels in primary Sjögren’s syndrome: an association with interstitial lung disease

**DOI:** 10.1186/s12890-024-02876-3

**Published:** 2024-02-07

**Authors:** You-Fan Peng, Fei-Yan Lu, Li-Ya Ma

**Affiliations:** 1 Department of Respiratory and Critical Care Medicine, The Affiliated Hospital of Youjiang Medical University for Nationalities, No.18 Zhongshan Er Road, 533000 Baise, Guangxi China; 2 Clinical Pathological Diagnosis and Research Centre, The Affiliated Hospital of Youjiang Medical University for Nationalities, Baise, China

**Retraction Note: BMC Pulmonary Medicine (2023) 23:366**

10.1186/s12890-023-02672-5

The Editor has retracted this article because, owing to an administration error in the editorial office, it was published before the peer review process had been undertaken. Post-publication peer review has concluded that the use of hospitalized patients rather than outpatients would have introduced confounding factors that may have affected bilirubin levels and their analysis. In addition, the inclusion of all variables in the both the univariate and multivariate analyses is incorrect. Therefore, after careful consideration, the Editor has taken the decision to retract the article given that if these issues had been highlighted by peer review before publication the article would not have been accepted. The Publisher apologises to the authors for the administrative error. The authors agree with this retraction.

